# TPEN/TPGS (T2) combo dramatically reduces Philadelphia chromosome-positive pro-lymphoblastic B leukemia in BALB/c mice

**DOI:** 10.1007/s12032-022-01873-y

**Published:** 2022-11-09

**Authors:** Miguel Mendivil-Perez, Marlene Jimenez-Del-Rio, Carlos Velez-Pardo

**Affiliations:** Neuroscience Research Group, Medical Research Institute, Faculty of Medicine, University of Antioquia (UdeA), Calle 70 No. 52-21, and Calle 62 # 52-59, Building 1, Room 412, SIU, Medellin, Colombia

**Keywords:** BALB-c, Ba/F3, BCR-ABL P210, Chronic leukemia, Combo, Neutropenia, Mice, TPEN, TPGS

## Abstract

Acute lymphoblastic leukemia (ALL) is hematological neoplasia that affects human beings from early life to adulthood. Although ALL treatment has been effective, an important percentage of ALL patients are resilient to treatment. Therefore, there is an urgent need for testing a new combination of compounds for the treatment of this disease. Recently, combined TPEN and TPGS (T2 combo) have shown selective cytotoxic effects in vitro leukemia cells such as Jurkat, K562, and Ba/F3 cells. In this study, we aimed to test the effect of combined TPEN and TPGS agents (T2 combo) at a fixed dose (TPEN 5 mg/kg: TPGS 100 mg/kg) on leukemic Ba/F3-BCR-ABL P210 BALB-c mice model. We found that 4 successive 2-day apart intravenous injections of T2 combo showed a statistically significant reduction of Ba/F3 BCR-ABL leukemia cells (− 69%) in leukemia BALB/c mice (*n* = 6) compared to untreated leukemia group (*n* = 6). Moreover, the T2 combo was innocuous to non-leukemia BALB/c mice (*n* = 3) compared to untreated non-leukemia mice (control, *n* = 3). After treatments (day 42), all mice were left to rest until day 50. Outstandingly, the leukemia BALB/c mice treated with the T2 combo showed a lower percentage of Ba/F3-BCR-ABL P210 cells (− 84%) than untreated leukemia BALB/c mice. Furthermore, treatment of leukemia and non-leukemia mice with T2 combo showed no significant tissue alteration/damage according to the histopathological analysis of brain, heart, liver, kidney, and spleen samples; however, T2 combo significantly reduced the number of leukocytes in the bone marrow of treated leukemia mice. We conclude that the T2 combo specifically affects leukemia cells but no other tissue/organs. Therefore, we anticipate that the T2 combo might be a potential pro-oxidant combination for the treatment of leukemia patients.

## Introduction

Acute lymphoblastic leukemia (ALL) is an aggressive liquid hematological tumor driven by malignant transformation and expansion of large numbers of immature T- and/or B-progenitor (T-ALL/B-ALL) lymphocytes [1, 2]. At diagnosis, both T-ALL and B-ALL are distinguished by the presence of  ≥ 20% blasts in the bone marrow [3]. Unfortunately, the Philadelphia chromosome (Ph)-a reciprocal translocation t(9;22)(q34;q11) leading to BCR-ABL fusion gene encoding BCR-ABL tyrosine kinase oncoprotein, is the most common cytogenetic abnormality in chronic myeloid leukemia (CML) as well as ALL, that increases with age i.e., 2–5% in childhood, 6% in adolescents and young adults, and more than 25% in adults [4]. Current treatment of ALL consists of high-intensity combination chemotherapy, e.g., Hyper-C (yclophosphamide) V (incristine) D (examethasone) A (driamycin), and tyrosine kinase inhibitors e.g., Imatinib [5, 6] resulting in high overall survival, with the best outcomes observed in pediatric patients [7]. Despite the high response rates after first-line therapy, about 20% of pediatric and 40% of adult patients will relapse [8]. Therefore, relapsed/refractory B-ALL treatment is an unmet need [9] and only a new combination of drugs/compounds (e.g., [10]) will have the potential to overturn the outcome of these patients.

Recently, our laboratory has shown that the T2 combo, composed of TPEN (a reactive oxygen species (ROS) generator agent and metal chelator), and TPGS (a synthetic derivative of natural vitamin E), induced  > 90% apoptosis at a ratio of TPEN 1:TPGS 20 in vitro Jurkat (clone-E61)-a model cell of ALL, K562-a model cell of chronic myeloid leukemia (CML), Ba/F3-a mouse pro-B-cells analogous to human B-ALL cells, and  > 75% apoptosis at similar ratio TPEN/TPGS in ex vivo acute pediatric acute B-cell patients leukemia cells [11]. However, whether the T2 combo is capable of selectively eroding leukemia cells in vivo is still not yet established.

To get insight into this issue, we have used cyclophosphamide-induced immunocompromised BALB/c mice and xenografted intravenously with (Philadelphia-positive) BCR-ABL Ba/F3 leukemia cells to test whether the T2 combo can ameliorate leukemic mice model. Here, we report that the T2 combo was effective to treat the leukemic mice model. Therefore, the T2 combo is potential in the treatment of ALL patients.

## Methods

### Mice

**Fig. 1:**
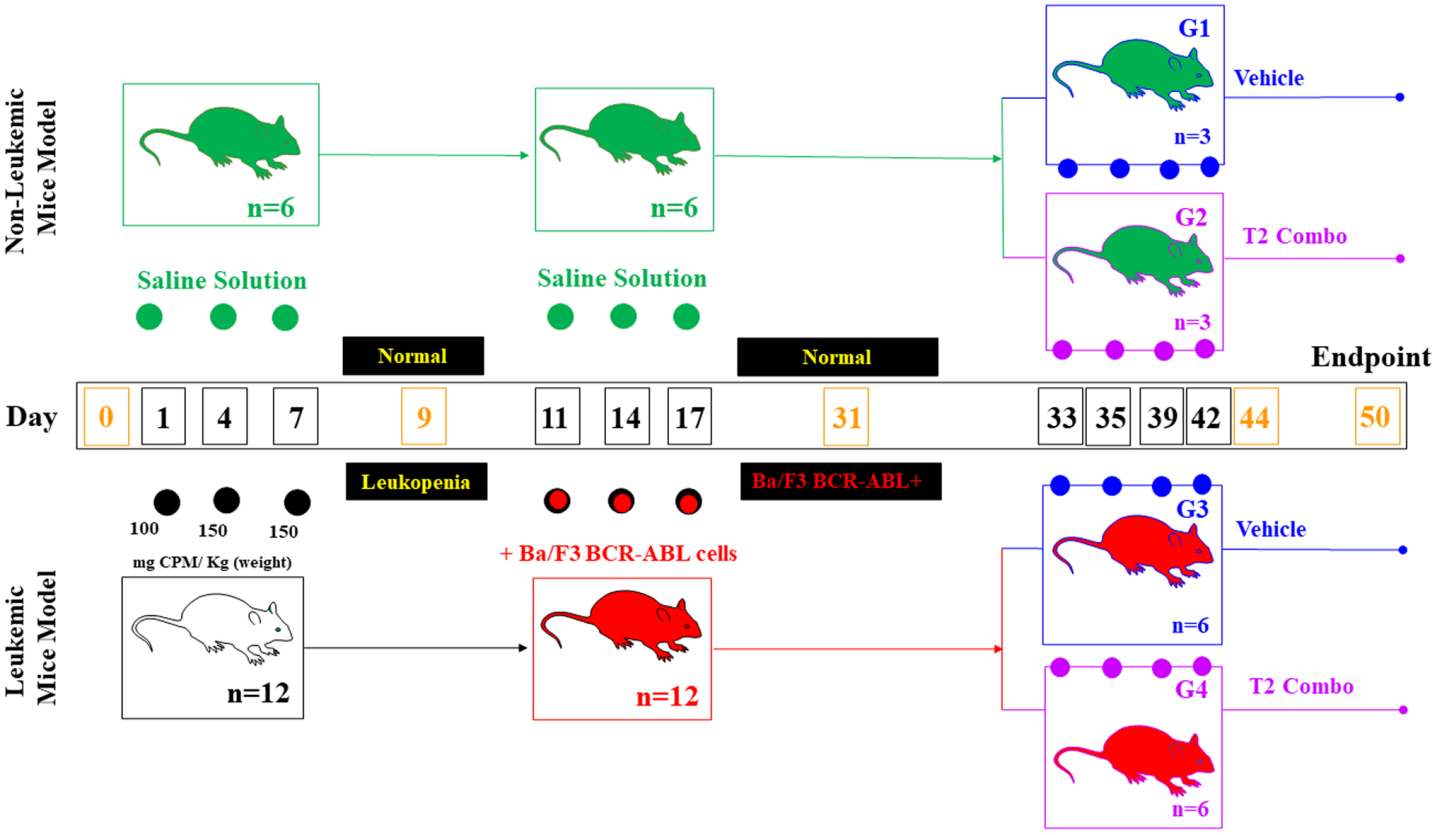
Timeline for leukemia induction and treatment. Schematic presentation of the cyclophosphamide-mediated immunological depletion protocol for induction of Philadelphia chromosome-positive pro-lymphoblastic B Leukemia in BALB/c mice and treatment schedule

All research procedures involving animals were according to and approved by the Ethical Committee for Animal Experimentation from Universidad de Antioquia (UdeA) Acta# 127 (2022/09/04). For all experiments, we used 7–9-week-old, male, non-specific pathogen-free BALB-c mice (Charles River, BALB/cAnNCrl, Strain Code 028, (https://www.criver.com/sites/default/files/resources/InbredMiceDatasheet.pdf), and weight 23–25 g. All mice received sterile food and water ad libitum and were housed in groups of three individuals during the experiments. The study was approved by the University of Antioquia Animal Care and Experimentation Committee (act #127-2019-09-04). Mice were initially allocated into two groups: non-cyclophosphamide treated mice (*n* = 6) and cyclophosphamide treated mice (*n* = 12). This last group of mice was xenografted with Ba/F3-BCR-ABL cells and labeled as leukemic mice. Then, non-leukemic mice (i.e., non-cyclophosphamide and non-xenografted mice) were treated with saline solution (SS, G1, *n* = 3) or treated with T2 combo i.e., TPEN (5 mg/kg)/TPGS (100 mg/kg) (G2, *n* = 3). Similarly, leukemic mice (i.e., cyclophosphamide treated and xenografted mice) were treated with SS (G3, *n* = 6) and T2 combo (G4, *n* = 6). The sample size of mice was calculated according to sample size calculator (https://clincalc.com/stats/samplesize.aspx; [12]) with the following variables: Study group design: two independent study group; primary endpoint: dichotomous (dead/not dead); statistical parameters: anticipated incidence group 1 (G1) = 1%, group 2 (G2) = 90%; enrollment ratio = 1; *α* = 0.05 (the probability of error type 1); *β* = 0.1 (the probability of error type 2); power = 0.90 (the ability to detect a difference between groups when a difference actually exists). As a result, groups 1 (G1) and 2 (G2) have a sample size of *n* = 3 each. For sample size of G3 and G4, we settled primary endpoint as dichotomous (leukemia/not leukemia), statistical parameters: anticipated incidence group 3 (G3) = 90%, group 4 (G4) = 15%; enrollment ratio = 1; *α* = 0.05; *β* = 0.2; power = 0.80. Therefore, G3 and G4 have a sample size of *n* = 6 each. A total of *n* = 18 mice were used (Fig. 1), thereby complying with the reduction (i.e., to use fewer animals in research) and refinement (i.e., to minimize discomfort in laboratory animals) principles of humane experimental (in vivo) technique [13].

### Cyclophosphamide (CPM) treatment

CPM treatment was performed according to Huyan et al. [14] with minor modifications. Briefly, CPM powder (Cytoxan^®^, Bristol Myers Squibb, NY, USA) was dissolved in distilled USP water for injection to a final concentration of 20 mg/mL. Twelve animals were subjected to CPM treatment. All mice received a total dose of 400 mg/kg by one 0.2 mL intraperitoneal injections (i.p.i.) scheduled at day 1 (100 mg/kg) and two 0.2 mL i.p.i. at day 4 and 7 (150 mg/kg) (Fig. 1).

### Blood leukocytes counts

Blood samples (~ 150 μl) were taken from the retroorbital plexus in heparinized capillary tubes (Modulohm A/S, Herlev, Denmark) at 9:00 AM on days 9, 31, 44, and 50 (Fig. 1). Total and differential white blood cell counts (neutrophils, lymphocytes, and monocytes) were performed manually for each sample using a Neubauer chamber (Brand GMBH, Wertheim, Germany) and microscopic examination of Wright-stained smears with 100X objective.

### Plasmid BCR-ABL P210 transfection in Ba/F3 cells, and cell culture conditions

The plasmid *BCR/ABL* P210-pLEF was a gift from Nora Heisterkamp (Addgene plasmid # 38158; http://n2t.net/addgene:38158; RRID: Addgene_38158, [15]), and used to transform Ba/F3 IL-3-dependent pro-B-cell line (Riken Bioresource Center-Cell Bank; RCB0805 cell line derived from C3H mouse strain) by nucleofection method using the Neon transfection system microporator (Cat# MPK 10096; Thermo Fisher Scientific). Briefly, 10 μg of purified plasmid BCR-ABL was electroporated in 7 × 10^6^ cells. For the transfection procedure, a pulse voltage of 1400 V, an amplitude of 20 ms, and 2 pulses were chosen. After transfection, the cells were cultured for 24 h in RPMI 1640 medium with IL-3 (10 ng/mL). Then, the cells that express BCR-ABL were grown at 37 °C in a humidified atmosphere of 5% CO_2_ in the air, in RPMI medium (SIGMA) supplemented with 10% FBS 100 U/mL penicillin and 100 μg/mL streptomycin (SIGMA) without cytokine IL-3 for 96 h. The Ba/F3 BCR-ABL P210 cells were used for further experiments.

### Xenografted Ba/F3-BCR-ABL cells-induced leukemia in BALB/c mice

Ba/F3 P210 BCR-ABL cells (5 × 10^6^ cells) were administered via intravenous (i.v.) injections into the lateral tail veins of immunosuppressed mice (*n* = 12), on days 11, 14, and 17 (Fig. 1), thereafter called leukemia mice (*n* = 12). To determine the presence of Ba/F3 BCR-ABL P210 cells in mice peripheral blood, we used the presence of both CD45 (a leukocyte marker, most expressed in lymphocytes) and F4/80 surface (https://cell.brc.riken.jp/en/rcb/baf3; Last update: 2022.07.25), and ABL proteins according to supplier’s recommendation. Briefly, total blood was collected, and 30 μl of the sample was incubated with rat anti-mouse FITC-CD45 (FITC anti-mouse CD45 Antibody, Cat# 103108, Biolegend), rat anti-mouse PE-F4/80 (PE anti-mouse F4/80 Antibody, Cat# 123110, BioLegend), and anti-ABL (c-Abl Antibody; Cat# MA5-14398; Invitrogen) antibodies for 15 min. Then, samples were incubated in red blood cell lysis solution for 30 min at 37 °C. Finally, the sample was centrifuged at 2000 rpm for 10 min. and incubated with DyLight 488 donkey anti-mouse antibody (1:500) (to identify ABL primary antibody), rinsed and resuspended for analysis on a BD LSR Fortessa II flow cytometer (BD Biosciences). Fifty thousand events were acquired, and the acquisition analysis was performed using FlowJo 7.6.2 Data Analysis Software.

### In vivo efficacy of TPEN/TPGS (T2 combo) in a mouse model of Ba/F3-BCR-ABL-induced leukemia

For the in vivo assessment of the T2 combo, 7–9-week-old males were administered via i.v. injections into the lateral tail veins (https://animalcare.ubc.ca/ available in 2022) with either saline solution (SS) to non-leukemia mice (G1, *n* = 3) and leukemic mice (G3, *n* = 6) or with T2 combo to non-leukemia mice (G2, *n* = 3) and leukemia mice (G4, *n* = 6, Fig. 1 on day 33, 35, 39, 42). The time scheduling of T2 combo treatment 4 successive 2-day apart i.v. injections in mice was opted according to [16–18]. Mice (G1–G4) were left to rest 8 days from day 42 to 50. Mice were observed daily for signs of stress (e.g., lethargy, ruffled coat, or ataxia) and changes in body weight to detect possible toxicities. Blood leukocytes and Ba/F3 BCR-ABL P210 cells were analyzed on days 31, 44, and 50 (orange color number in Fig. 1) for a peripheral blood smear test, hemogram analysis, and flow cytometry. Day 50 was the experimental endpoint (Fig. 1).

### BCR-ABL immunofluorescence analysis

To determine the BCR-ABL reactivity we evaluated the ABL positive cells in cell cultures and bone marrow smears. Briefly, cells were fixed with cold ethanol (− 20 °C) for 20 min., followed by 10% bovine serum albumin (BSA) blockage. Then after, cells were incubated overnight with anti-ABL mouse monoclonal antibody 1:200 followed by exhaustive rinsing and incubation with DyLight 488 donkey anti-mouse antibody (1:500). The nuclei were stained with 1 µM Hoechst 33,342 (life technologies).

### BCR-ABL western blotting analysis

Ba/F3 cells (1 × 10^7^) were left non-transfected or transfected as described above and then whole cells were lysed in 50 mM Tris–HCl, pH 8.0, with 150 mM sodium chloride, 1.0% Igepal CA-630 (NP-40), and 0.1% sodium dodecyl sulfate, 1 nM PMSF and a protease inhibitor cocktail (Sigma-Aldrich). Then, 40 μg of proteins in reducing loading buffer were loaded onto 6% electrophoresis gels and transferred to nitrocellulose membranes (Hybond-ECL, Amersham Biosciences) for 10 min using an electrophoretic transblot system (BIO-RAD). The membranes were incubated overnight at 4 °C with monoclonal mouse anti-ABL antibody (see above). We used mouse anti-actin (1:1000, cat #MAB1501, Millipore) as an expression control. IRDye 680CW donkey anti-mouse (LI-COR Biosciences; 1:10,000) were used as the secondary probe. The blots were developed using the Odyssey Infrared Imaging System. The WB analysis includes three lectures from independent transfection experiments. We used K562 lysates as BCR-ABL positive control.

### Photomicrography and image analysis

Light microscopy photographs were taken using an Olympus BX53 microscope equipped with an Olympus DP74 camera. The fluorescent microscopy photographs were taken using a Zeiss Axiostart 100 Fluorescence Microscope equipped with a Zeiss AxioCam Cm1 (Zeiss Wöhlk-Contact-Linsen, Gmb Schcönkirchen, Germany). Images were analyzed by ImageJ software (http://imagej.nih.gov/ij/). The figures were transformed into 8-bit images and the background was subtracted. The cellular measurement regions of interest (ROI) were drawn over cell and the fluorescence intensity or cell area were subsequently determined by applying the same threshold for controls and treatments.

### Tissue processing

Tissue biopsies (e.g., brain, kidney, liver, heart, spleen, and bone marrow) were collected on day 50 (endpoint, Fig. 1) and fixed in 10% neutral-buffered formaldehyde and embedded in paraffin. Paraffin-embedded tissues were sectioned into 3- to 4-µm serial sections and processed for gross histopathology by hematoxylin–eosin staining according to histological standard procedures (e.g., https://www.biolegend.com/en-us/protocols/immunohistochemistry-protocol-for-paraffin-embedded-sections), and the tissues were examined under a microscope. Tissue assessment was performed at the laboratory of Animal Pathology, Diagnostic Unit of the Faculty of Agricultural Sciences, UdeA.

### Statistical analysis

Statistical analyses were performed using the GraphPad Prism 6 scientific software (GraphPad, Software, Inc. La Jolla, CA, USA). Student’s *t*-test, one-way or two-way ANOVA with a Tukey post hoc test was used to compare the differences between the experimental groups. For graphical display of data, a *box and whisker plot* were used. A *P*-value < 0.05 (*), < 0.01 (**) and < 0.001 (***) were statistically significant.

## Results

### Xenografted Ba/F3-BCR-ABL cells in immunosuppressed BALB/c mice induce leukemia

**Fig. 2:**
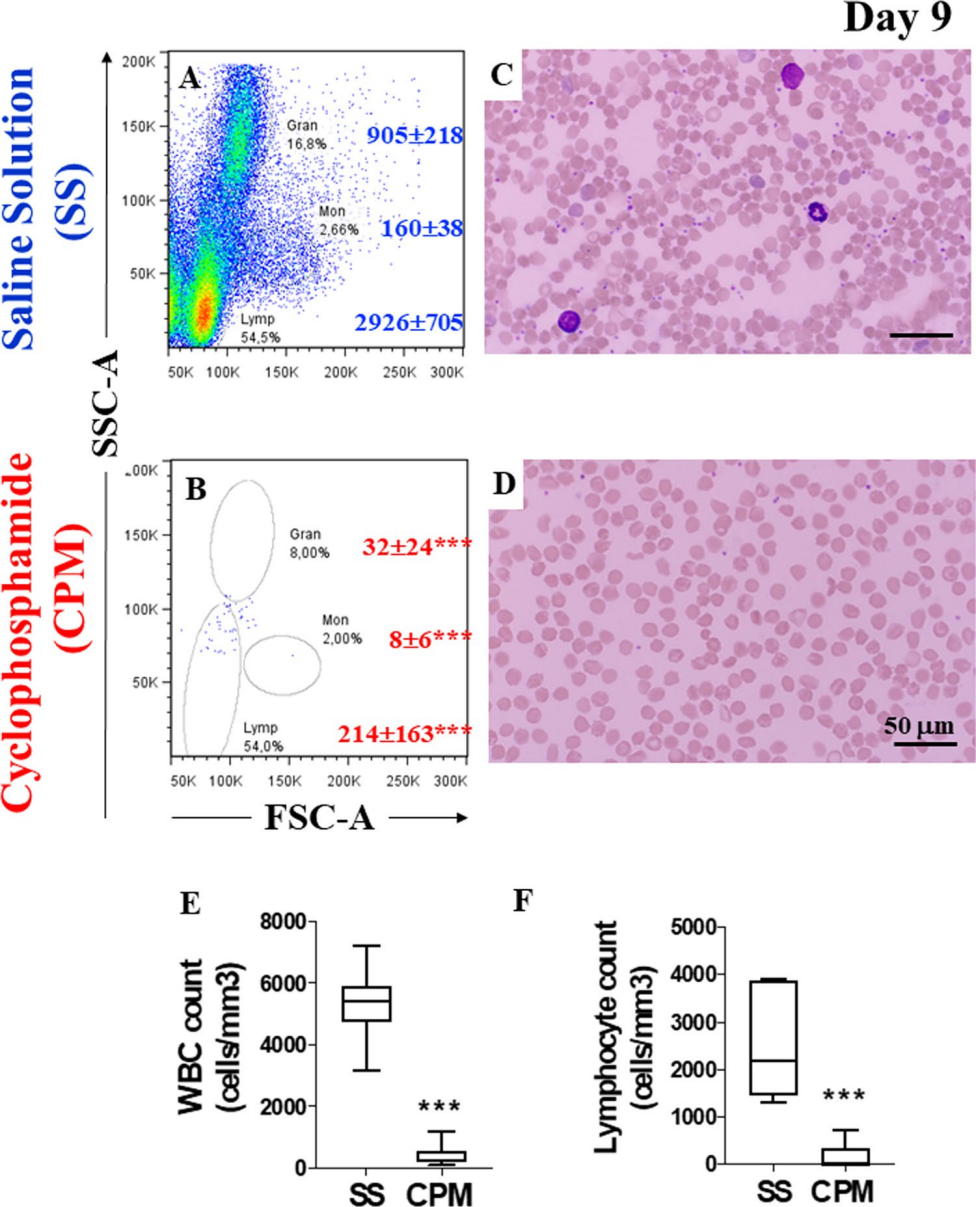
Cyclophosphamide (CPM) immunosuppressed BALB/c mice. **A** Peripheral blood flow cytometry analysis of leukocyte subpopulation counts and percentage from mice treated with Saline Solution (SS, *n* = 6), **B** or Cyclophosphamide (CPM, *n* = 12). **C** Wright staining of blood smear from mice treated with SS or **D** CPM. **E** Peripheral blood cell counts from SS and CPM groups analyzed for WBC, and **F** lymphocytes. Significant values were determined by Student’s *t*-test; ***p < *0.001*. Image magnification × 100

We first wanted to generate a reliable leukemic mice model. Previous studies indicated that two intraperitoneal i.p. injections of cyclophosphamide (CPM) at 150 mg/kg at 2-day intervals may establish good immunosuppressive models of BALB/c mice for studying the fungal pathogenicity [14]. Therefore, a group of BALB/c mice was treated with saline solution (SS, *n* = 6) or treated with 100, 150, and 150 mg cyclophosphamide (CPM)/kg (*n* = 12) on days 1, 4, and 7, respectively (Fig. 1). On day 9, flow cytometry analysis of peripheral blood leukocytes from mice treated with SS or CPM revealed 2 different profiles. Mice treated with SS presented typical cell subpopulations easily distinguished based on cell size (forward scatter) and complexity (side scatter) such as granulocytes (17%, 905 ± 218 cells, absolute counts), monocytes (3%, 160 ± 38) and lymphocytes (55%, 2926 ± 705 cells, Fig. 2A), whereas mice treated with CPM showed almost no leukocyte subpopulations (Fig. 2B, granulocytes 32 ± 24; monocytes 8 ± 6, lymphocytes 214 ± 163). Similar observations were obtained by hemogram (Fig. 2C) and blood smear analysis (Fig. 2D, E, F), thereby confirming that mice under CPM exposure effectively were immunosuppressed.

**Fig. 3:**
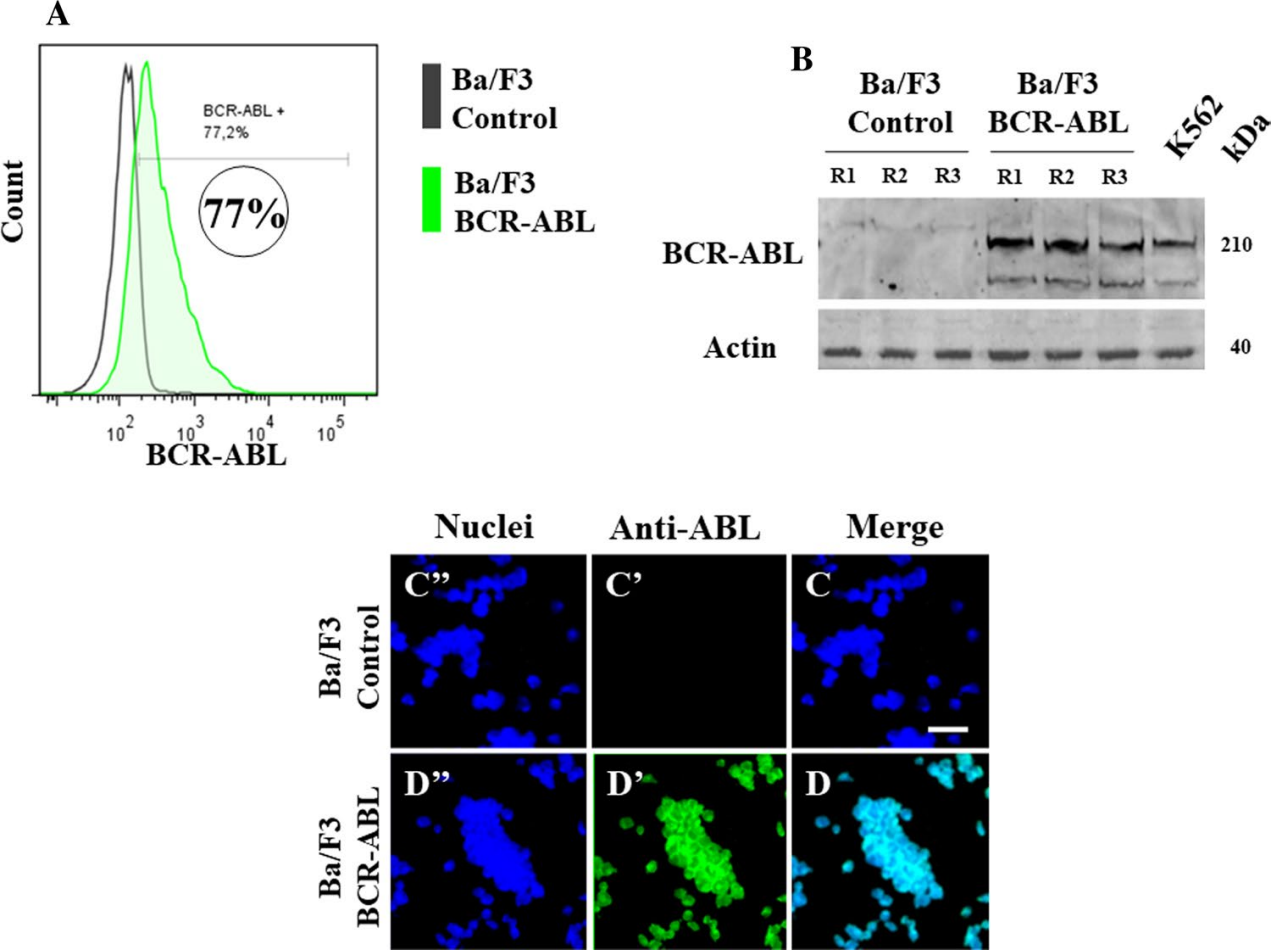
Establishment of Philadelphia chromosome-positive pro-lymphoblastic Ba/F3 cells. **A** Flow cytometry analysis showing Ba/F3 cells transfection efficiency in transfected (Ba/F3 BCR-ABL, green histogram) and non-transfected (Ba/F3 control, black histogram) cells. **B** Western blot analysis of 210 kDa *BCR-ABL* fusion protein in transfected (Ba/F3 BCR-ABL) and non-transfected (Ba/F3 control) cells, compared to K562 cells lysate (positive control). Immunofluorescence analysis showing (**C**′′–**D**′′) the nuclei staining, (**C**′–**D**′)**,** the ABL reactivity, and **C**, **D** the merge images in transfected (Ba/F3 BCR-ABL) and non-transfected (Ba/F3 control) cells

Since Ba/F3 is an IL-3-dependent pro-B-cell line amenable to be transformed into an IL-3-independent pro-B-cell line by expression of oncogenic kinase BCR-ABL protein [19, 20], we used plasmid *BCR/ABL* P210-pLEF to transform naïve Ba/F3 cells. As shown in Fig. 3, the nucleofection transfection method of the plasmid into Ba/F3 cells showed a high transfection efficiency of about 77% (Fig. 3A). Further analysis by Western blot positively identified the 210 kDa band representing the molecular weight of the oncogenic kinase protein BCR-ABL in transfected Ba/F3 cells but absent in non-transfected cells (Fig. 3B). These observations were confirmed by the immunofluorescence technique (Fig. 3C versus D).

**Fig. 4:**
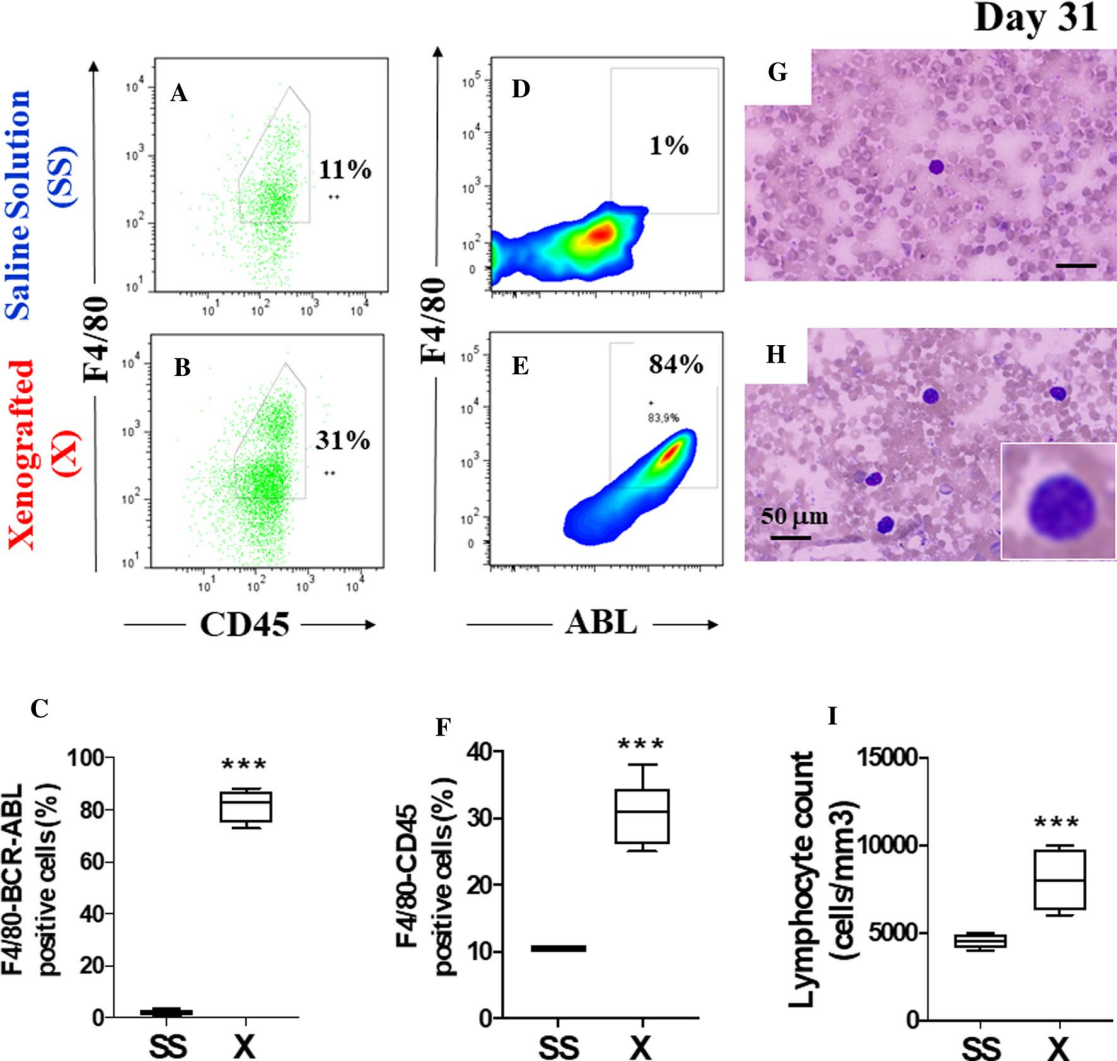
*Ba/F3-BCR-ABL* cells-induced leukemia in immunosuppressed xenografted mice. **A** Representative images showing flow cytometry analysis of F4/80^+^/CD45^+^ double positive cells from the Saline Solution (SS), and **B** Xenografted mice (X) groups. **C** Quantitative data showing the mean percentage analysis of F4/80^+^/CD45^+^ double positive cells from SS (*n* = 6) and X (*n* = 12) groups. **D** Representative images showing flow cytometry analysis of F4/80^+^/ABL^+^ double positive cells from the SS and **E** X groups. **F** Quantitative data showing the mean percentage analysis of F4/80^+^/ABL^+^ double positive cells from SS and X groups. **G** Wright staining of blood smear from mice treated with SS or **H** X groups. **I** Peripheral blood lymphocyte counts from SS and X groups. Significant values were determined by Student’s *t*-test; ***p < *0.001*. Image magnification ×100. Inset magnification ×500

We then proceeded to xenograft *Ba/F3-BCR-ABL* cells into immunosuppressed BALB/c mice (*n* = 12) on days 11, 14, and 17 (Fig. 1), and treated in parallel a group of non-xenografted mice (*n* = 6) with SS (Fig. 1). After 14 days post-cell transfusion (day 31), flow cytometry analysis evidenced that non-xenografted mice exhibited a significantly low percentage of F4/80-CD45 (Fig. 4A–C) and F4/80 ABL positive cells (Fig. 4D, F) compared to xenografted mice (Fig. 4B–F). Naturally, the xenografted mice showed a higher percentage of F4/80-CD45 marker e.g., 31% of which 84% were F4/80 ABL positive cells compared to non-grafted mice (e.g., 11% F4/80-CD45 of which 1% was F4/80 ABL positive cells). Lymphocyte counts were significantly reduced in non-xenografted mice compared to xenografted mice (Fig. 4I) according to in blood smear assessment (Fig. 4G, H). Together these data indicated that xenografted *Ba/F3-BCR-ABL* mice were genuinely leukemic mice model.

### TPEN/TPGS (T2) combo induces a dramatic reduction of Ba/F3-BCR-ABL cells in the leukemic mice model

**Fig. 5:**
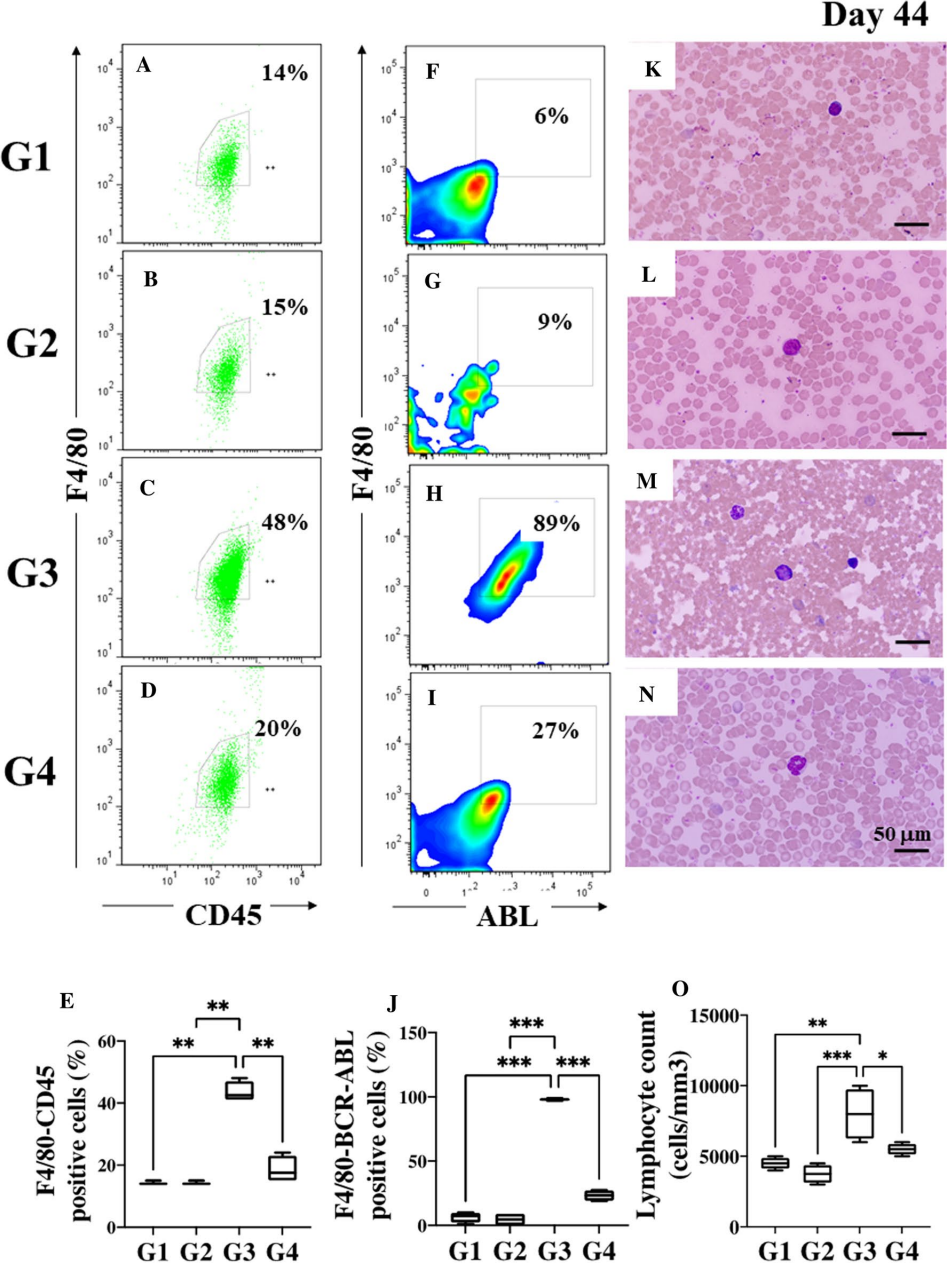
TPEN/TPGS (T2) combo induces a dramatic reduction of Ba/F3-BCR-ABL cells in the leukemic mice model. **A** Representative images showing flow cytometry analysis of F4/80^+^/CD45^+^ double positive cells from the non-leukemic mice treated with SS (G1; *n* = 3), **B** non-leukemic mice treated with T2 combo (G2; *n* = 3), **C** leukemic mice treated with SS (G3; *n* = 6) and **D** leukemic mice treated with T2 combo (G4; *n* = 6). **E** Quantitative data showing the mean percentage analysis of F4/80^+^/CD45^+^ double positive cells from G1–G4. **F** Representative images showing flow cytometry analysis of F4/80^+^/ABL^+^ double positive cells from non-leukemic mice treated with SS (G1), **G** non-leukemic mice treated with T2 combo (G2), **H** leukemic mice treated with SS (G3) and **I** leukemic mice treated with T2 combo (G4). **J** Quantitative data showing the mean percentage analysis of F4/80^+^/ABL^+^ double positive cells from G1–G4. **K** Wright staining of blood smear from non-leukemic mice treated with SS (G1), **L** non-leukemic mice treated with T2 combo (G2), **M** leukemic mice treated with SS (G3) and **N** leukemic mice treated with T2 combo (G4). **O** Peripheral blood lymphocyte counts from G1–G4. Significant values were determined by one-way ANOVA with a Tukey post hoc test; *p < 0.05; **p < 0.005 ***p < *0.001*. Image magnification ×100

Next, we investigated whether TPEN/TPGS combo [11] was capable to suppress Ba/F3-BCR-ABL cell population in vivo. To this aim, four sub-groups of mice were obtained from SS and xenografted mice: group G1 (non-leukemic mice, *n* = 3) and G2 (non-leukemic mice, *n* = 3) were treated with SS and T2 combo, respectively, at 2-day intervals four times (Fig. 1, day 33, 35, 39 and 42), and G3 (leukemic mice, *n* = 6) and G4 (leukemic mice, *n* = 6) were also treated with SS and T2 combo at similar 2-day intervals four times as described. On day 44, cytometry analysis showed that the T2 combo was innocuous to both non-leukemic mice (G1) and non-leukemic (G2) according to F4/F8-CD45 (Fig. 5A vs B) and F4/80 ABL (Fig. 5F vs G) markers. In contrast, the T2 combo dramatically reduced Ba/F3-BCR-ABL cells in leukemic mice (G4) compared to untreated leukemic mice (G3) according to a significant difference between F4/F8-CD45 (Fig. 5C vs D, E) and F4/80 ABL (Fig. 5H vs I, J) markers. Clearly, of the 20% leucocytes and Ba/F3-BCR-ABL cells in G4 plus T2 combo, at least 27% were Ba/F3-BCR-ABL cells, whereas, of the 48% leucocytes and Ba/F3-BCR-ABL cells in untreated G3, 89% were Ba/F3-BCR-ABL cells. Interestingly, the T2 combo reduced-69% Ba/F3-BCR-ABL cells in G4 mice. Similar results were obtained by blood smear analysis (Fig. 5K–N) wherein lymphocytes count in the G4 plus T2 combo tended to almost normal values as G1 (Fig. 5O).

### TPEN/TPGS (T2) combo is still active after 8 days of post-treatment in leukemia mice

**Fig. 6:**
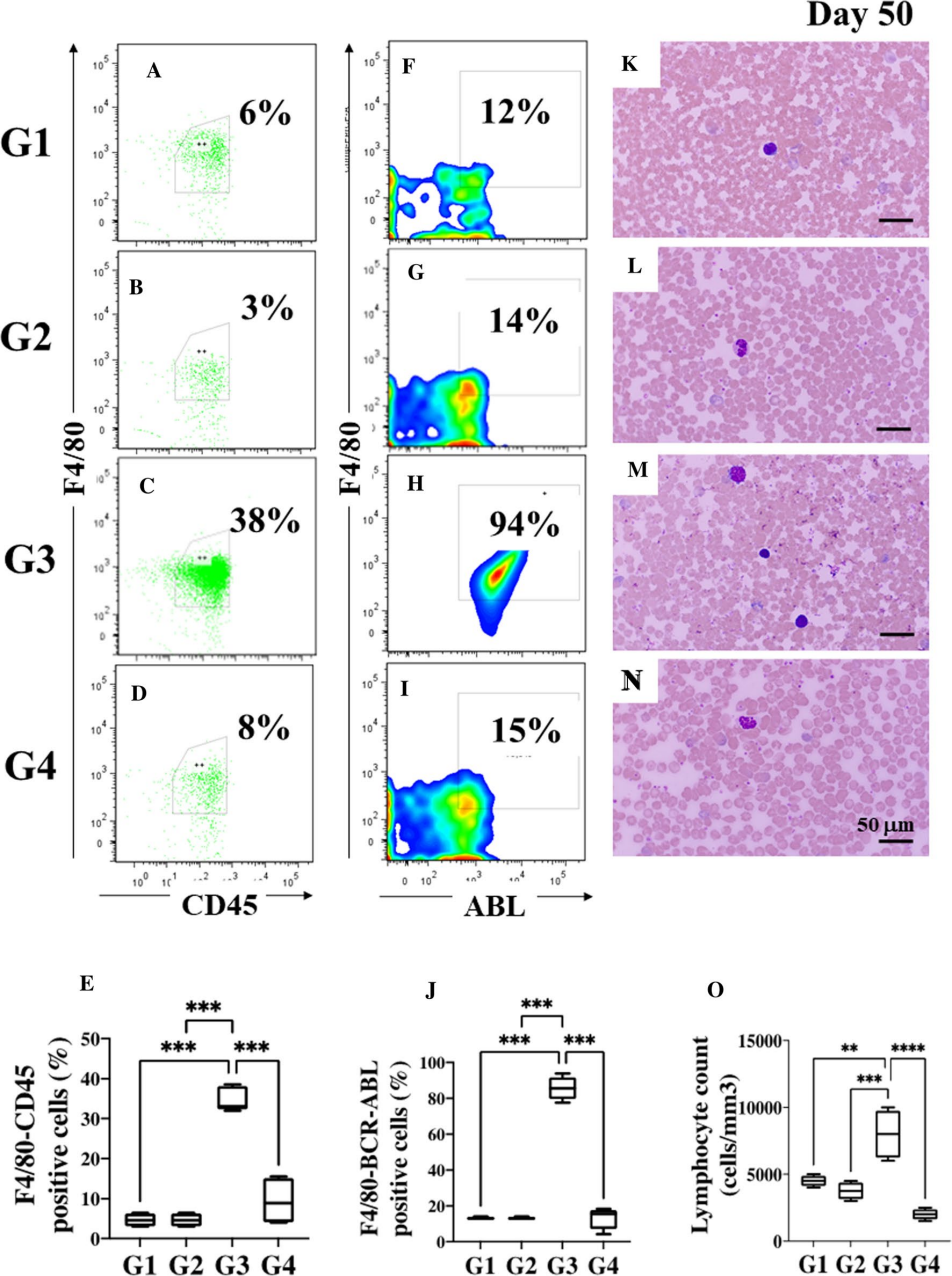
TPEN/ TPGS (T2) combo is still active after 8 days of post-treatment in Leukemia mice. **A** Representative images showing flow cytometry analysis of F4/80^+^/CD45^+^ double positive cells from the non-leukemic mice treated with SS (G1; *n* = 3), **B** non-leukemic mice treated with T2 combo (G2; *n* = 3), **C** leukemic mice treated with SS (G3; *n* = 6) and **D** leukemic mice treated with T2 combo (G4; *n* = 6). **E** Quantitative data showing the mean percentage analysis of F4/80^+^/CD45^+^ double positive cells from G1–G4. **F** Representative images showing flow cytometry analysis of F4/80^+^/ABL^+^ double positive cells from non-leukemic mice treated with SS (G1), **G** non-leukemic mice treated with T2 combo (G2), **H** leukemic mice treated with SS (G3) and **I** leukemic mice treated with T2 combo (G4). **J** Quantitative data showing the mean percentage analysis of F4/80^+^/ABL^+^ double positive cells from G1–G4. **K** Wright staining of blood smear from non-leukemic mice treated with SS (G1), **L** non-leukemic mice treated with T2 combo (G2), **M** leukemic mice treated with SS (G3) and **N** leukemic mice treated with T2 combo (G4). **O** Peripheral blood lymphocyte counts from G1–G4. Significant values were determined by one-way ANOVA with a Tukey post hoc test; **p < 0.005 ***p < *0.001*. Image magnification × 100

We wonder whether after 8 days of post-treatment (day 42–50 in Fig. 1), TPEN/TPGS combo continues to operate against Ba/F3-BCR-ABL cells in leukemia mice. Figure 6 shows that though no significant changes were observed in lymphocytes population counts from G1 (Fig. 6A, E, F, J, K, and O), and G2 (Fig. 6B, E, G, J, L, and O) according to flow cytometry and blood smear assessment, T2 combo reduced − 84% Ba/F3-BCR-ABL cells in G4 mice according to F4/80 ABL (Fig. 6I, J) compared to G3 (Fig. 6H, J). These results were confirmed by blood smear observations wherein lymphocyte count in G4 was significantly reduced (Fig. 6N, O) compared to G3 (Fig. 6M, O).

### TPEN/ TPGS (T2) combo induces a dramatic reduction of Ba/F3-BCR-ABL cells in bone marrow from leukemia mice

**Fig. 7:**
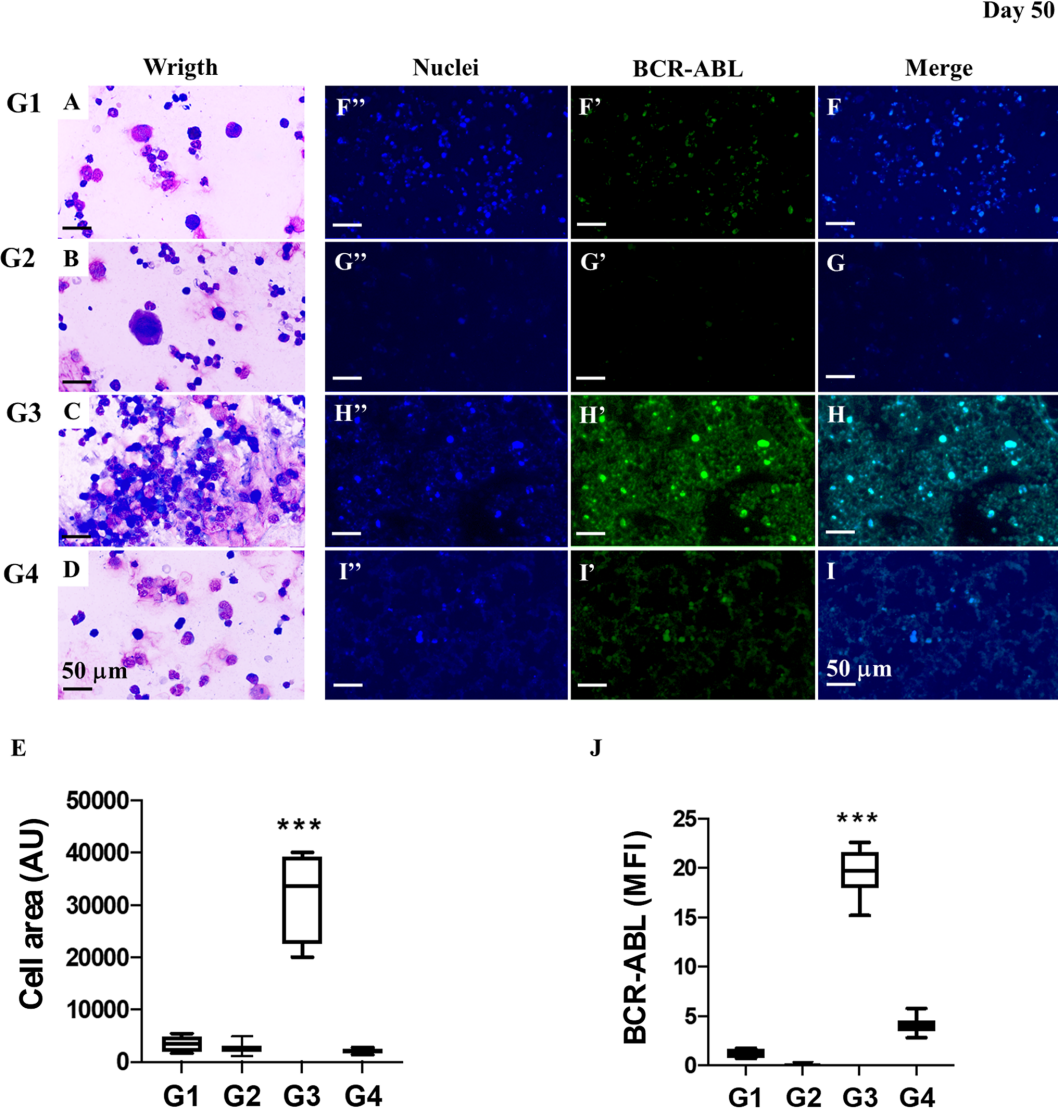
TPEN/ TPGS (T2) combo induces a dramatic reduction of Ba/F3-BCR-ABL cells in bone marrow from leukemia mice. **A** Wright staining of bone marrow smear from non-leukemic mice treated with SS (G1; *n* = 3), **B** non-leukemic mice treated with T2 combo (G2; *n* = 3), **C** leukemic mice treated with SS (G3; *n* = 6), and **D** leukemic mice treated with T2 combo (G4; *n* = 6). **E** Quantitative data showing the leukocyte cells area in BM from G1–G4. Representative pictures showing (**F**′**′**–**I**′′) nuclei, (**F**′–**I**′) BCR-ABL and (**F**–**I**) merge images from (**F**′′–**F**) non-leukemic mice treated with SS (G1), (**G**′′–**G**) non-leukemic mice treated with T2 combo (G2), (**H**′′–**H**) leukemic mice treated with SS (G3), and (**I**′′–**I**) leukemic mice treated with T2 combo (G4). (**J**) Quantitative data showing the BCR-ABL mean fluorescence intensity in BM from G1-G4. Significant values were determined by one-way ANOVA with a Tukey post hoc test; *p < 0.05; **p < 0.005 ***p < *0.001*. Image magnification ×100

We further investigated the effect of the T2 combo on bone marrow (BM) in leukemia mice. As shown in Fig. 7, the values of leukocyte cells area in BM from untreated G1 (Fig. 7A) and treated G2 (Fig. 7B) showed no statistical differences according to cell area in Wright stain (Fig. 7E). In contrast, T2 combo induced a significant reduction in the number of cells per area in BM from treated G4 (Fig. 7D, E) compared to untreated G3 (Fig. 7C, E). To confirm these observations, BM smears were investigated for the presence of oncogenic BCR-ABL marker by immunofluorescence. While lymphocyte cells from G1 (Fig. 7F, J) and G2 (Fig. 7G, J) expressed almost no protein BCR-ABL in BM, there was a significant increase in the amount of BCR-ABL positive in BM from G3 (Fig. 7H, J) but almost none BCR-ABL positive were recorded in G4 treated with T2 combo (Fig. 7I, J).

### TPEN/TPGS (T2) combo causes no harm to vital organs in leukemic mice model

**Fig. 8:**
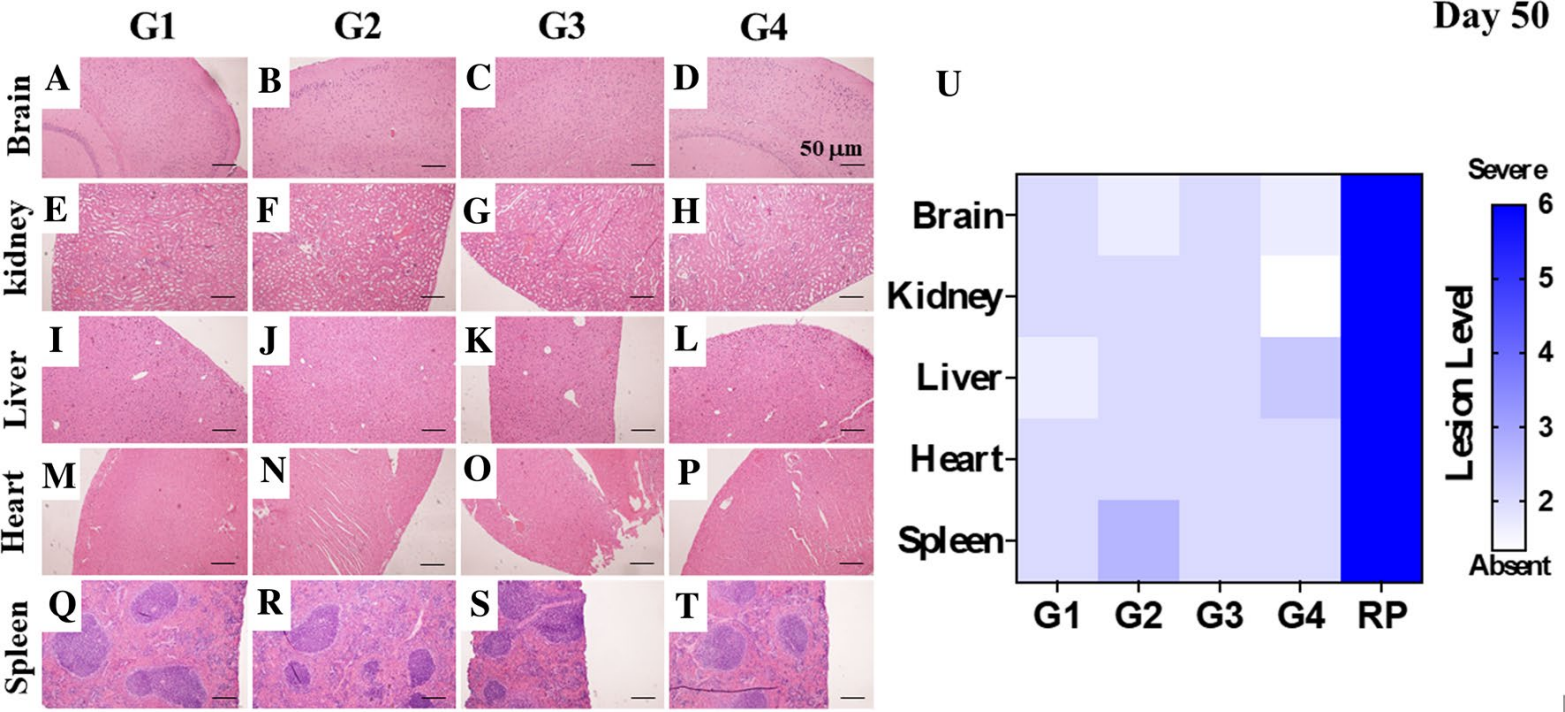
TPEN/TPGS (T2) combo is harmless to vital organs in leukemic mice model. Tissue histopathology showing H&E staining of brain (**A**–**D**), kidney (**E**–**H**), liver (**I**–**L**), heart (**M**–**P**) and spleen (**Q**–**T**) from (**A**, **E**, **I**, **M**, **Q**) non-leukemic mice treated with SS (G1; *n* = 3), (**B**, **F**, **J**, **N**, **R**) non-leukemic mice treated with T2 combo (G2; *n* = 3), (**C**, **G**, **K**, **O**, **S**) leukemic mice treated with SS (G3, *n* = 6), and (**D**, **H**, **L**, **P**, **T**) leukemic mice treated with T2 combo (G4; *n* = 6). **U** Heat map analysis of tissue lesion intensity [scale 0–6 compared to severe lesion reference pattern (RP)] Significant values were determined by two-way ANOVA with a Tukey post hoc test; *p < 0.05; **p < 0.005 ***p < *0.001*. Image magnification ×100

Lastly, we inquired whether the T2 combo might be detrimental to vital organs in both non-leukemia and leukemia mice treated with the T2 combo. As shown in Fig. 8, the histopathological analysis revealed no tissue damage in the brain (Fig. 8A–D), kidney (Fig. 8E–H), liver (Fig. 8I–L), heart (Fig. 8M–P), or spleen (Fig. 8Q–N) from untreated G1 (Fig. 8U) or treated G2 (Fig. 8U), and untreated G3 (Fig. 8U) or treated G4 (Fig. 8U).

## Discussion

The present investigation was undertaken to evaluate the therapeutic effect of the TPEN/TPGS (T2) combo in a leukemic mice model. Here, we report for the first time that the T2 combo was highly effective in reducing the xenografted Philadelphia chromosome-positive (BCR-ABL) pro-lymphoblastic Ba/F3 cells in immunocompromised BALB/c mice. Several observations support this assumption. First, we found that 4 successive 2-day apart intravenous injections of T2 combo at a fixed dose (TPEN 5 mg/kg: TPGS 100 mg/kg) showed a statistically significant reduction of Ba/F3 BCR-ABL leukemia cells (– 69%) in leukemia BALB/c mice compared to untreated leukemia group. Furthermore, the effectiveness of the T2 combo in reducing the percentage of Ba/F3 BCR-ABL leukemia cells even decreased to − 84% after 8 days post-treatment. These results merit some consideration. *(i) T2 ratio *Previous in vitro and ex vivo data have shown that a ratio TPEN 1: TPGS 20 induced  > 90% and  > 75% apoptosis in Jurkat cells as well as in Ba/F3 cells [11], we, therefore, adopted T2 combo dose (TPEN 5 mg/kg: TPGS 100 mg/kg) at the same ratio (1:20). This dose proved to be safe and non-toxic per se to mice, as evidenced in G2 mice who survived unharmed to T2 treatment. *(ii) Route of T2 infusion *Although pharmacokinetic (PK) studies are not yet available for TPEN, it has recently been demonstrated that TPGS (5 mg/kg) intravenous administration, but not oral administration, in rats was rapidly distributed to the spleen, liver, lung, and kidney before being slowly eliminated in urine and feces [21]. Therefore, increasing the TPGS concentration (e.g., to 100 mg/kg or 20-times relative to Run et al. study [21]) might favor that this agent might be readily available to affect leukemia cells. The same logic might not be operative for TPEN because low doses of TPEN (≤ 10 mg/kg) have been reported to be well tolerated in mice, whereas high concentrations e.g., 20 mg/kg has led to ataxia and loss of coordination but ≥ 30 mg/kg has led not only to ataxia, loss of coordination, convulsions but also to death in 20.3 min or less [22]. This last observation has been confirmed by others (e.g., [23]), however, 15–20 mg/kg have been reported to be well tolerated [23] suggesting that response to TPEN (10–20 mg/kg range) might depend on mice strain. *(iii) Time of treatment* Compared to other experimental treatments e.g., a combination of vincristine (0.5 mg/kg) and AMD11070 (10 mg/kg) lasted 7 weeks after US.7 cells (1.5 × 10^6^ cells per mouse) transplant [24], our treatment approach is relatively short, since it lasted 11 days (first leukemia evaluation), or 17 days (second leukemia evaluation) after xenograft Ba/F3 BCR-ABL leukemia cells (5 × 10^6^ cells per mouse). We conclude that the high effectiveness of the T2 combo combating leukemia cells in mice might be attributed to its route of infusion (e.g., i.v.i.), dose (ratio 1:20), and the bioavailability of their components, and short time of treatment. These considerations are critical if the T2 combo might be applied to human leukemia patients. Second, we found that the T2 combo was not only highly effective against peripheral leukemia cells but also highly effective in eroding them in bone marrow (BM) in the leukemic mice model. Interestingly, leukemia mice treated with T2 combo (G4) reached a similar number of cells per area as untreated non-leukemia mice (G1) or non-leukemia mice treated with T2 combo (G2) in BM. These observations suggest that both TPEN and TPGS can reach tissues with low blood perfusion rates (e.g., bone marrow) specifically destroying malignant cells. This feature makes the T2 combo of special attention pharmaceutically because BM is a tissue where most of the leukemic stem cells reside, and blast cells stop further development, thereby protecting leukemia cells against the cytotoxicity of chemotherapeutic agents and becoming a possible source of relapse. Moreover, BM has been implicated as a privileged microenvironment in resistance to leukemia therapy [25]. The specific activity of the T2 combo in BM and blood makes this combination highly effective to treat leukemia systematically. Lastly, the T2 combo was ineffective harming vital organs/tissues including brain, heart, liver, kidney, and spleen samples. This implies that the T2 combo is capable of specifically discriminating between non-malignant and malignant cells. Taken together these observations suggest that the T2 combo is safe for vital organs and specifically toxic to leukemia cells. Since TPEN and TPGS alone or in combo have demonstrated antileukemia activity in vitro [11, 26, 27] and in vivo (this work), T2 combo support the view that increasing intracellular ROS is an excellent therapeutic strategy for battling leukemia cells [28–30]. Therefore, the T2 combo might be considered as a promising pro-oxidant anticancer duet.

No less important, we developed an acute lymphoblastic B leukemia mice model. We found that compared to other methodologies to obtain leukemic mice models e.g., xenograft mouse models involving genetically manipulated immunodeficient mice (e.g., Poster: Immunodeficient Oncology Mouse Models: https://www.criver.com/resources/info-pi-rm-immunodeficient-mouse-models-charles-river-na, [31]), genetically engineered mouse models (e.g., [31–33]) or ex vivo transduction transplantation models wherein malignant cells are injected in irradiated recipient animals [34], our leukemia model is simple, economic, time-saving, and reliable that reproduce the basic feature of leukemia, i.e., overgrowth of blast cells in blood and bone marrow. Indeed, we took advantage of the following data. First, the BALB/c mouse is the most common inbred model used in experimental laboratories worldwide. Specifically, BALB/cAnNCrl Strain (Code 028, http://www.informatics.jax.org/strain/MGI:2683685) shows normal innate immunity and is used for general multipurpose applications, therefore amenable to chemically induced immunosuppression. Second, cyclophosphamide is a potent immunosuppressive agent currently used for in vivo research (e.g., [14, 35]), it is pharmacologically well characterized [36] and is commercially available. Third, Ba/F3 is a murine interleukin-3-dependent pro-B-cell line that can be easily transformed into an interleukin-3 independent responsive cell line. Indeed, ectopic expression of an oncogenic e.g., BCR-ABL, could faithfully recapitulate B-ALL in mice. Both the transfection of vector BCR-ABL P210-pLEF (15,850 bp vector size) by nucleofection was highly efficient (> 70%) and transformed Ba/F3 cells growth and BCR-ABL expression were easily detected in vivo by flow cytometry and Western blot technique, respectively. Altogether, we were able to develop a leukemic Ba/F3-BCR-ABL mice model in about 4 weeks (31 days). However, it is noteworthy to mention that, thought nursing care cost maintenance is high and time-consuming, a more straightforward leukemic mice model can be established using BALB/c nude mice, which appears to be an immunocompromised mouse strain, and the transplantable human chronic myeloid leukemia K562 cells, which express BCR-ABL (e.g., [37]). Based on our previous findings [11], we anticipate that the T2 combo will be able to regress leukemia cell charge in the leukemic K562 BALB/c nude mice model.

## Conclusion

Currently, treatment with combination of several cytotoxic agents and prolonged therapy is essential for cure. Despite those efforts, an important percentage of the patients do not respond to chemotherapy and relapse. T2 combo has demonstrated highly efficient in the treatment of pro-B-cell leukemia in mice eroding about 70% of leukemia cells by 4 successive 2-day apart infusions, or 84% after 8 days post-treatment. Further studies may inquire into the effects of more prolonged treatment. Altogether, the T2 combo proved to be safe, not toxic for vital organs, and specifically lethal for leukemia cells in peripheral blood and bone marrow. Although PK studies are known for TPGS [21], this information is not yet available for TPEN. Nonetheless, the T2 combo is a promising therapeutic alternative for those patients for whom standard chemotherapy has failed.

## Data Availability

All relevant data and materials are within the paper.
